# Supplementary material for: Deep genome annotation of the opportunistic human pathogen Streptococcus pneumoniae D39
Source: Nucleic Acids Res. 2018 Aug 13;46(19):9971–89. doi: 10.1093/nar/gky725 (PMC6212727; doi:10.1093/nar/gky725)
Supplement: Supplementary Data [file gky725_supplemental_files.zip › Supplementary material_revised.docx]

**SUPPLEMENTARY FIGURES**

**
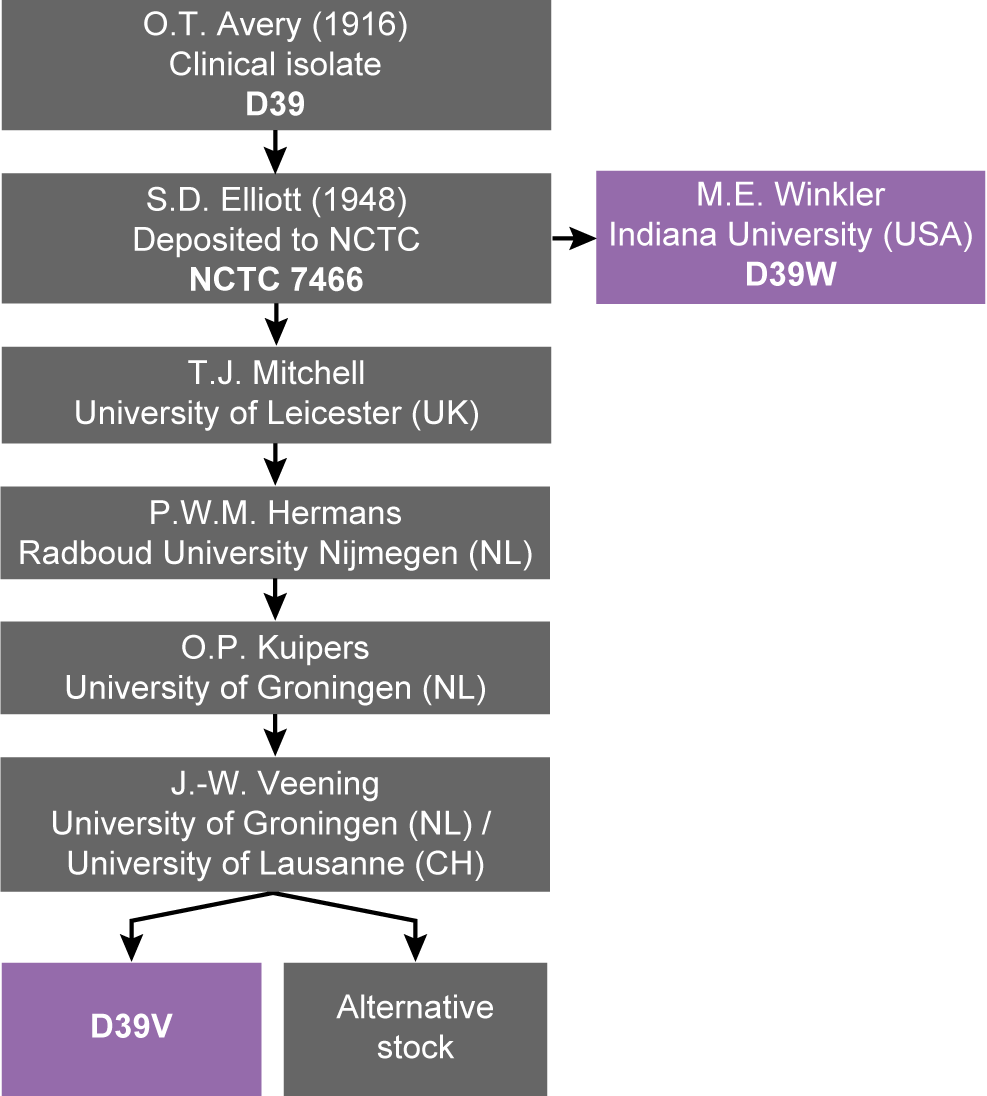
**

**Figure S1.** History of D39V. D39 was isolated in 1916 by Avery and submitted to the National Collection of Type Cultures (NCTC) in 1948. From there, cells have been recultured several times before reaching the Veening lab (D39V).

**
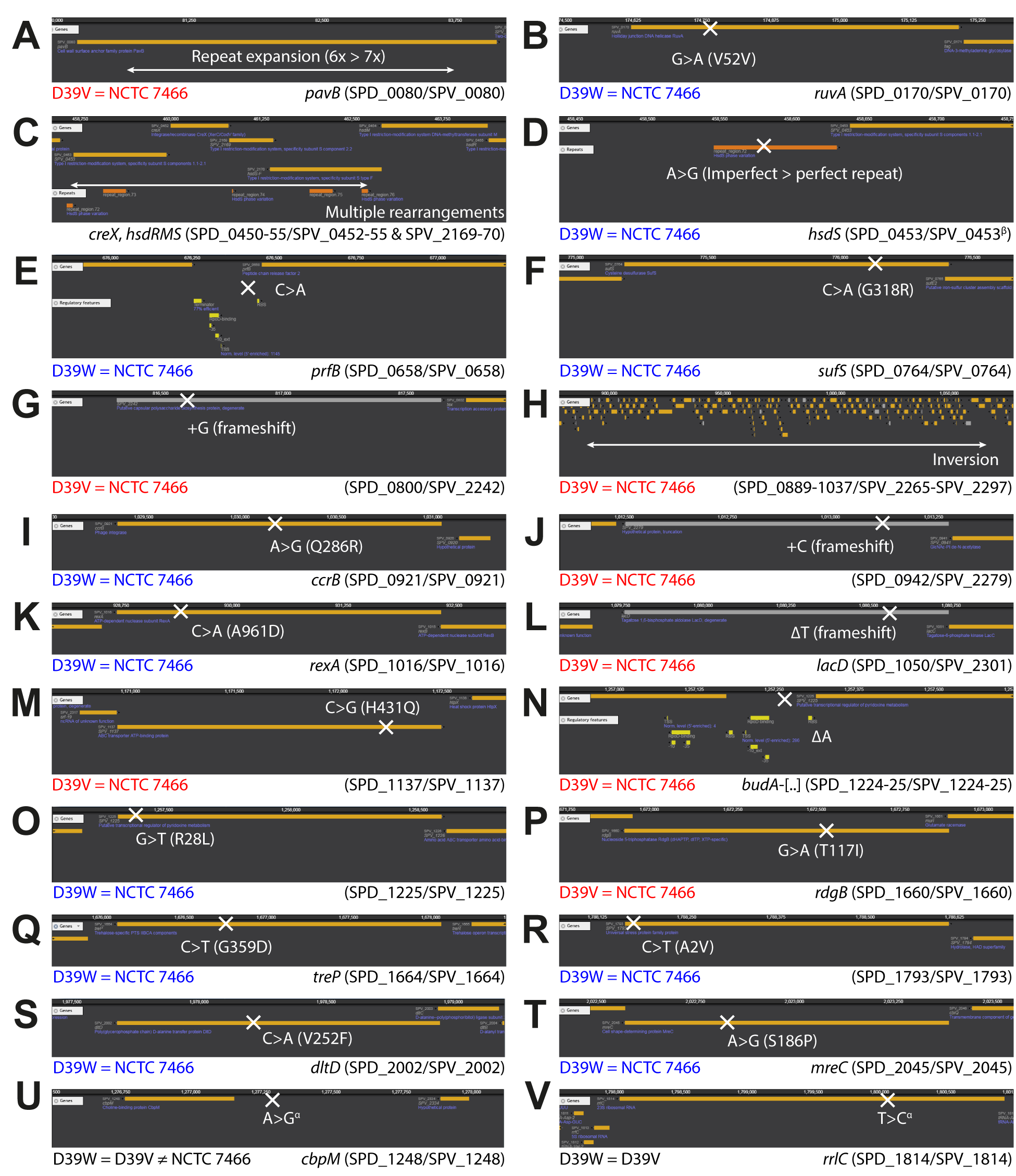
Figure S2.** Genomic context in D39V of sites of differing sequence with D39W (CP000410). A-V. Regions containing sequence differences between D39W and D39V (A-T) and between D39W/D39V and NCTC 7466 (U-V), matching the order in main Table 1. The exact location of observed differences and their consequences, based on the situation in the D39W assembly, are indicated in white. The strain matching the ancestral NCTC 7466 is indicated below each panel. ^α^Difference relative to NCTC 7466. ^β^Inside rearranged region, probably not transcribed.

**
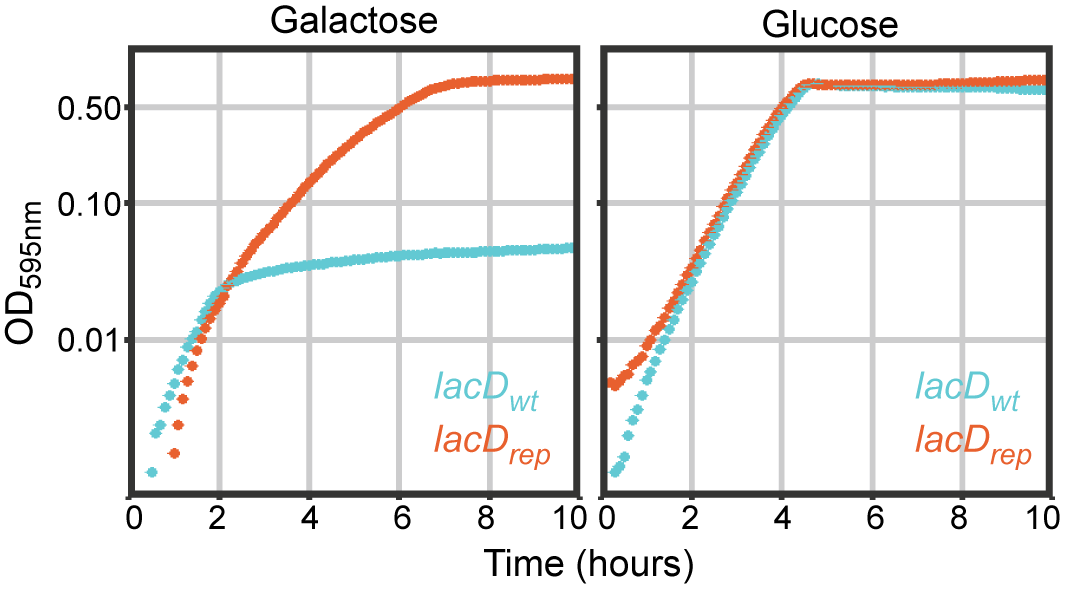
**

**Figure S3.** Restored growth on galactose of a *lacD*-repaired strain. Wild-type D39V (*wt*) and a mutant carrying the intact R6 allele of *lacD* (*rep*), were grown in a plate reader in C+Y medium with either galactose (left) or glucose (right) as primary carbon source. As expected, repairing *lacD* restored growth on galactose.


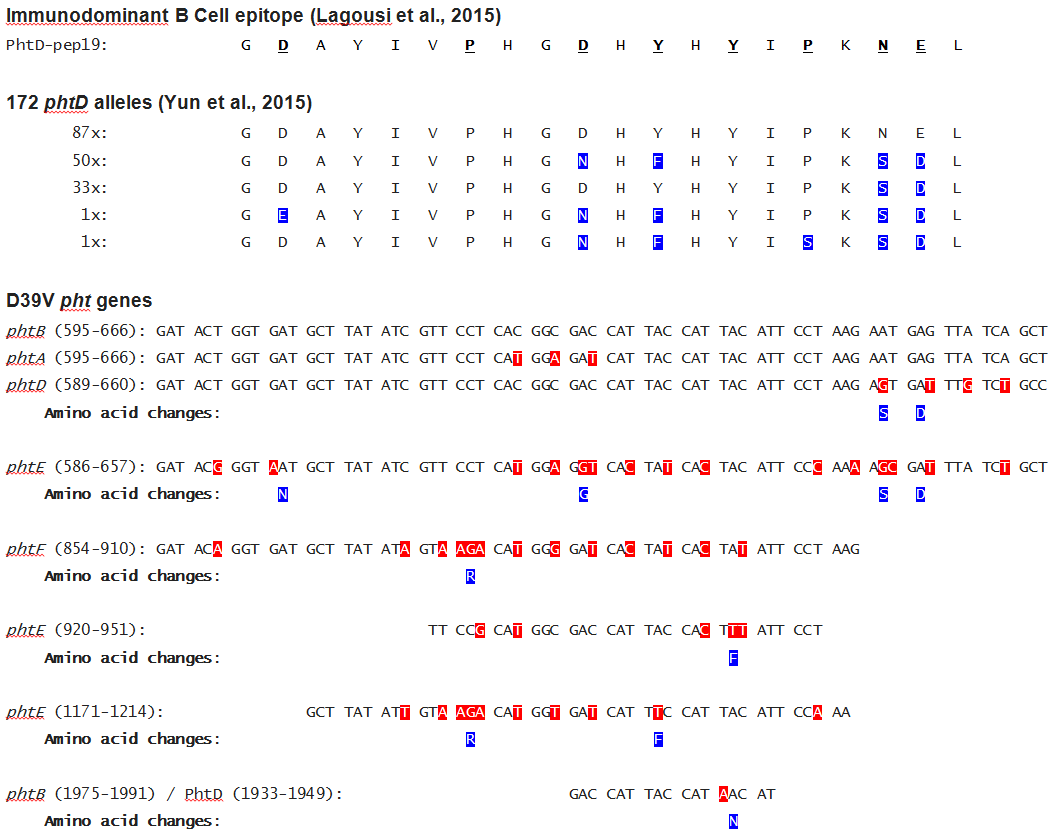


**Figure S4.** PhtD variation potential; sequence analysis of a PhtD region (PhtD-pep19) reported to have the highest reactivity with serum from patients suffering from invasive pneumococcal disease (1). Amino acid changes in 6 different locations are observed in the 172 *phtD* alleles sequenced by Yun et al. (2). Multiple sequence alignment of D39V *pht* genes reveals the possibility of four additional changes in case of intrachromosomal recombination events.

**
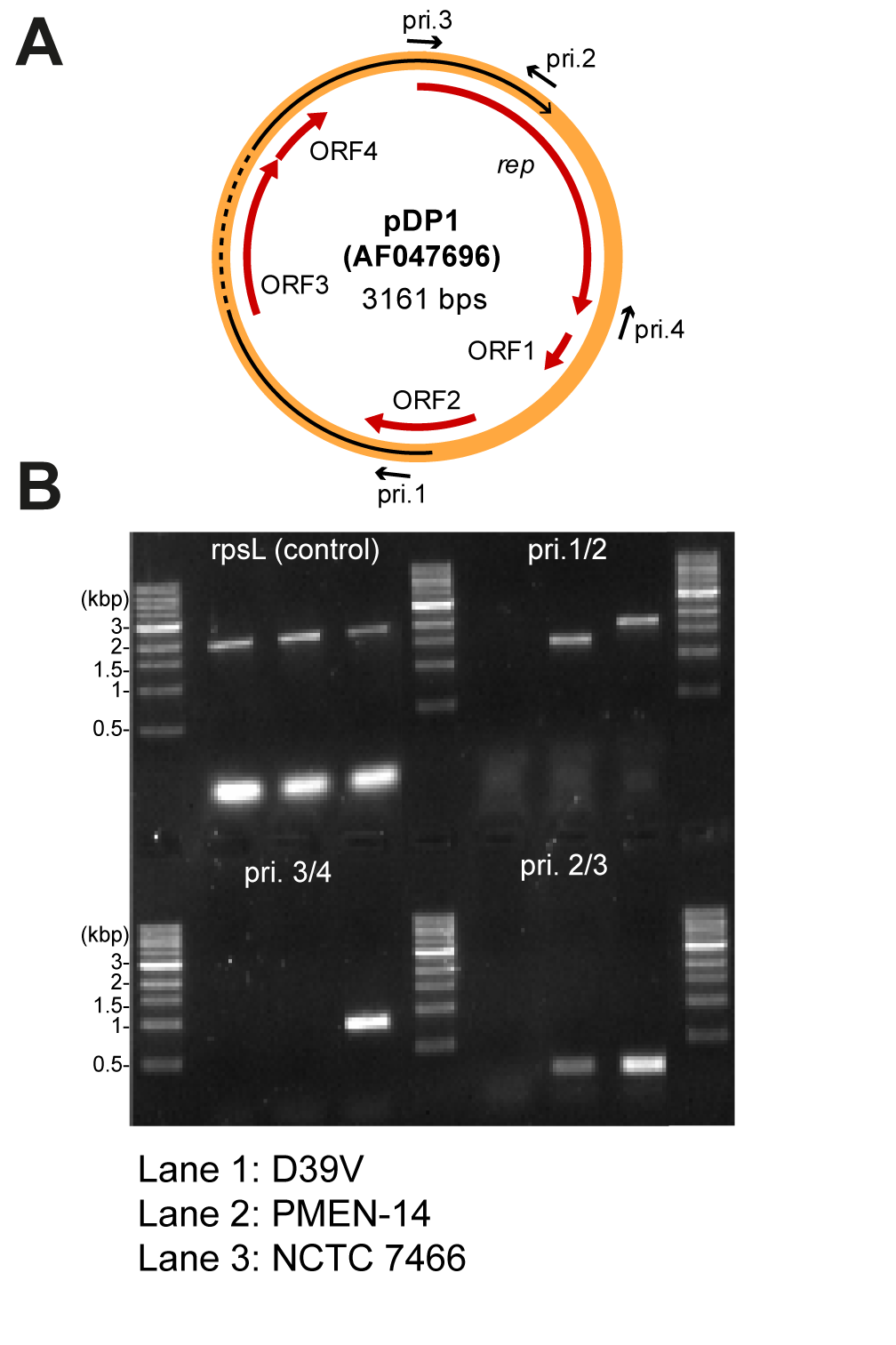
**

**Figure S5.** PCR-based assay on presence or absence of cryptic plasmid pDP1. A. Organization of plasmid pDP1 (orange), including binding sites of primers used for amplification. The black line indicates how part of the plasmid was integrated in strain PMEN-14. B. Electrophoresis gel after DNA amplification with 4 primer pairs. Lanes 1-3: D39V, PMEN-14 and NCTC 7466. Positive control (*rpsL*) gives the correct product for all samples. All expected products are observed for both NCTC 7466 samples and for PMEN-14, which has a degenerate version of the plasmid integrated into its chromosome. The absence of product in all of the D39V assays confirms that this strain lacks plasmid pDP1.


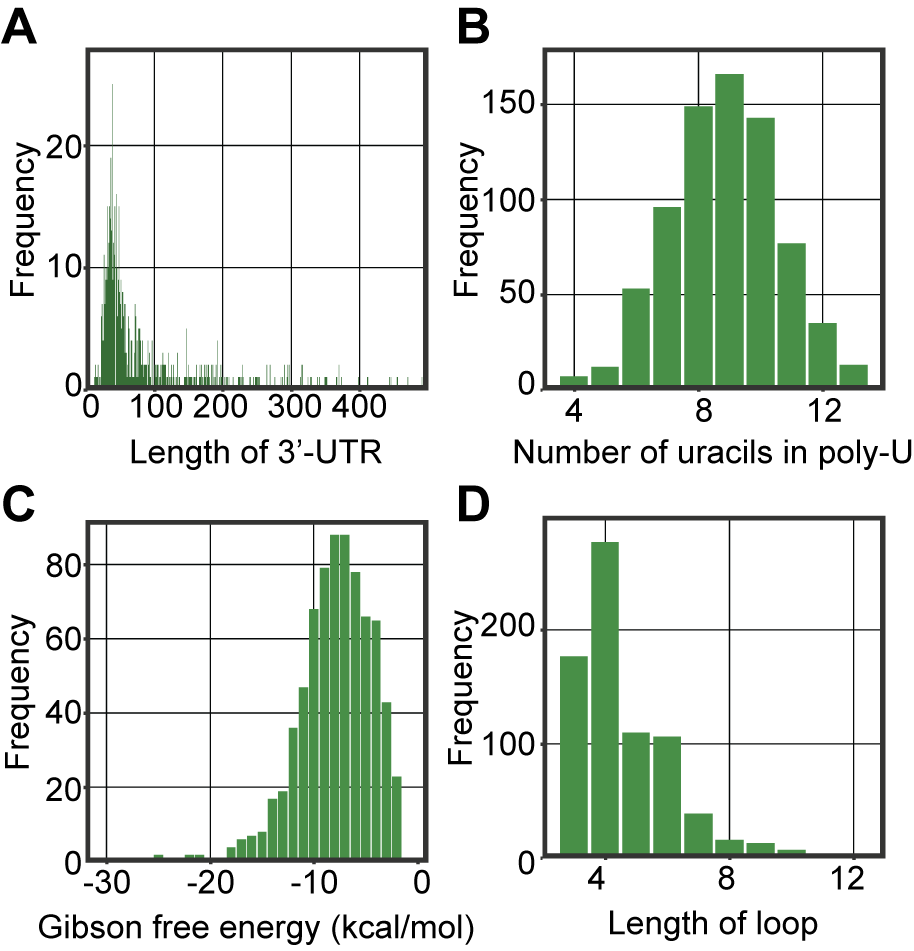


**Figure S6.** Characteristics of annotated terminators. Only terminators were included that both contained a predicted stem-loop (TransTermHP, (3)) and a detected termination peak. A. Distribution of terminator distance to the first feature upstream (3’-UTR). B. Frequency distribution of the number of uracils in a 15-nucleotide window following the predicted stem-loop structure. C. Binding energy distribution of the stem sequence, as reported by TransTermHP. D. Length distribution of the loop between both arms of the terminator stem.

**SUPPLEMENTARY METHODS**

**Culturing of *Streptococcus pneumoniae* D39 and transformation**

*S. pneumoniae* strains were routinely grown at 37°C, diluted 1:100 from -80°C stock into C+Y (pH 6.8), a liquid casein-based medium, supplemented with yeast extract (Sigma-Aldrich, Boom, NL), as described in (4).

Construction of strains was performed as previously described (5). For transformation, cells were grown until OD_600nm_ ~0.1. Subsequently, 100 ng·ml^-1^ synthetic CSP-1 (competence-stimulating peptide-1) was added into the suspension and incubation was continued for another 12 min at 37°C. Transforming DNA was added to the now-competent *S. pneumoniae*. Cells were then incubated at 30°C for 20 min to allow for DNA internalization. Afterwards, fresh C+Y was added and the suspension was incubated further at 37°C for 90 minutes. Selection of transformants was done by plating in Columbia agar (Oxoid, UK) supplemented with 2% sheep blood (Johnny Rottier, NL) and 0.50 μg·ml^-1^ tetracycline for *luc-gfp* constructs or 0.25 mg·ml^-1^ kanamycin for *luc* constructs. The following day, colonies were streaked

on antibiotic-supplemented blood agar and incubated overnight in 5% (v/v) CO_2_. Constructs were checked by colony PCR and Sanger sequencing.

**Strain construction**

*Transcriptional fusions of pyrimidine operons and* luc. Strains (RA59, RA60, RA61, RA62, RA63, RA65, RA68, RA69) were constructed in which *luc* was inserted downstream of each of eight different pyrimidine metabolism operons (*pyrG*, *pyrFE*, *pyrDa*-*holA*, *pyrH*-*frr*, *pyrKDb*, *ung*-*mutX*-*pyrG*, *pyrRB*-*carA*, *uraA*). For each operon, upstream and downstream fragments were PCR-amplified from genomic DNA, using primer pairs [last_gene]_up_F / [last_gene]_up_R_BglII and [last_gene]_down_F_NotI / [last_gene]_down_R, respectively. The cassette carrying *luc* and a kanamycin resistance marker was amplified from strain MK134 with primer pair Luc_F_BglII_AseI_EcoRI / kan-R+NotI. Fragments were cut with BglII and NotI and ligated together. Subsequently, wild-type D39V cells were transformed with the ligation product and positive clones were selected on kanamycin and verified by Sanger sequencing.

*Transcriptional fusions of* *P*_pbp2a_ *and* luc. Three strains were constructed in which *luc* was controlled by P*_pbp2a_* in an ectopic locus (*bgaA*). In RA114, the leader of *luc* contained just the first three codons of *pbp2a*. Plasmid pLA18 (*luc*_*gfp*) was amplified with primer pair G033 / G044, while P*_pbp2a_* was amplified with G035 / G036. In RA115, the *luc* leader also contained an RBS. Plasmid pLA18 was amplified with G037 / G034, while P*_pbp2a_* was amplified with G035 / G038. In RA122, the *luc* leader contained the first three codons of *pbp2a* followed by a stop codon. Plasmid pLA18 was amplified with G039 / G034, while P*_pbp2a_* was amplified with G035 / G040. For each strain, both fragments were assembled using Gibson’s assembly (6) and the resulting molecule was used to transform wild-type D39V cells. Positive clones were selected on tetracycline and verified by Sanger sequencing.

*Strain with repaired* lacD. To repair the observed frameshift in *lacD*, we replaced the D39V allele of *lacD* with the intact version of the gene from *S. pneumoniae* R6. The latter gene was amplified from R6 cells with primer pair lacD_F / lacD_R and the PCR product was used to transform D39V cells. Positive clones were identified by growth on galactose and further verified by Sanger sequencing.

**Growth, luciferase and GFP assays**

Before the assays, cells were routinely pre-cultured in C+Y medium until an OD_600_ of 0.4, and then diluted 1:100 into fresh medium in a 96-wells plate. All assays were performed in a Tecan Infinite 200 PRO at 37°C.

Luciferase assays were performed in C+Y with 0.25 mg·ml^-1^ D-luciferin, sodium salt and signals were normalized through division by OD_595_.

To normalize fluorescence signals, we included a parental strain without *gfp* and used the growth-dependent autofluorescence readings of that strain to subtract from fluorescence signals of *gfp*-containing strains.

Growth assays of *lacD*-repaired strains were performed in C+Y with either 10.1 mM galactose or 10.1 mM glucose as main carbon source.

**DNA and RNA isolation, primary transcript enrichment and sequencing**

Wild-type *S. pneumoniae* cells were pre-cultured in C+Y medium and diluted, 1:100, into 100 ml fresh C+Y medium. Cells were harvested by centrifugation at an OD_600_ of 0.4. Isolation of genomic DNA was performed using the Wizard Genomic DNA Purification Kit (Promega), where the nuclei lysis buffer was supplemented with 0.05% SDS, 0.025% sodium deoxycholate and 200 mg/ml RNase A. DNA was further purified by ethanol precipitation. A total of 12 μg genomic DNA was sent to the Functional Genomics Center Zurich, where a 6/8 kbp insert library was created with a lower cut-off of 4kbp and was then sequenced using a PacBio RS II machine. Supporting Illumina HiSeq DNA-seq data was obtained previously as described (7, 8).

D39V samples for RNA-seq were pre-cultured in suitable medium before inoculation (1:100) into four infection-relevant conditions: lung-like medium, CSF-like medium, fever-like conditions in CSF-like medium and late competence (20 min after CSP addition) in C+Y medium. Composition of media, a detailed description of conditions and the total RNA isolation protocol are described in the accompanying paper by Aprianto et al. (4). Isolated RNA was sent to vertis Biotechnologie AG. Total RNA from the four conditions was combined in an equimolar fashion and the pooled RNA was divided into two portions. The first portion was directly enriched for primary transcripts (Cappable-seq (9)) and, after library preparation according to the manufacturer’s recommendations, sequenced on Illumina NextSeq in single-end (SE) mode. This technique exploits the fact that primary transcripts carry a 5’-triphosphate group, while processed transcripts carry a monophosphate group. The second RNA portion was rRNA-depleted and sequenced on Illumina NextSeq in paired-end (PE) mode, to obtain information about both 5’ and 3’ ends of sequenced fragments.

***De novo* assembly of the D39V genome and DNA methylation analysis**

*De novo* genome assembly was performed using the Hierarchical Genome Assembly Process (HGAP3) module of the PacBio SMRT portal version 2.3.0. This resulted in two contigs: one of over 2 Mbp with 250-500x coverage, and one of 12 kbp with 5-25x coverage. The latter, small contig was discarded based on its low coverage and high sequence similarity with a highly repetitive segment of the larger contig. The large contig was circularized manually, by removing a 14 kbp sequence that occurred both at the start and end of the sequence. The resulting sequence was verified by re-mapping the data using the ‘RS_Resequencing.1’ module and then rotated such that *dnaA* was positioned on the positive strand, starting on the first nucleotide. Previously published Illumina data (GEO accessions GSE54199 and GSE69729) were mapped on the new assembly, using *breseq* (10). The datasets lacked coverage in the *bgaA* locus, because the sequenced strains were *bgaA*::P*_ssbB_*-*luc*. This locus, together with four loci of potential mistakes in the assembly were verified by Sanger sequencing. Only one actual mistake was identified in the *de novo* assembly.

DNA methylation analysis was performed using the ‘RS_Modification_and_Motif_Analysis.1’ module in the SMRT portal, with a QV cut-off of 100: firstly, methylated bases are identified based on the knowledge that the presence of such modifications significantly affects the polymerization rate in a well-characterized fashion (11). Afterwards, the sequence context of all detected modifications is analyzed for the enrichment of certain sequence motifs (12), results of which are reported in **Figure 2C**.

**Detection of cryptic plasmid pDP1**

To evaluate whether an analogous plasmid to pDP1 was present in D39V, we performed a PCR-based assay. Amplification products were obtained for NCTC 7466 cells, with primer pairs pDP1.1F / pDP1.2R; pDP1.3F / pDP1.4R; and pDP1.3F / pDP1.2R. The absence of these products when using D39V as a template suggested that no such plasmid existed in this strain. As an extra validation step, we mapped RNA-seq data to the DNA sequence of pDP1, using Bowtie 2 (13). When no reads mapped to pDP1, we concluded that the plasmid was indeed absent from D39V.

**Automated and curated annotation**

The assembled genome sequence was annotated automatically, using PGAP ((14), executed October) 2015) and RAST ((15), executed June 2016). The results of both annotations were compared and for each discrepancy, support was searched. Among the support used were scientific publications (PubMed), highly similar features (BLAST (16)), reviewed UniProtKB entries (17) and/or detected conserved domains as found by CD-Search (18). When no support was found for either the PGAP or RAST annotation, the latter was used. Duplicate gene names were also resolved during curation. After the first round of curation, the resulting annotation was compared with the curated annotation of R6 (NC_003098.1) to complete the working annotation by identifying loci potentially mis-annotated by RAST and PGAP. R6 is a D39 derivative that shares many identical features. Again, the R6 annotation was only adopted in the presence of convincing support. Finally, an extensive literature search was performed, with locus tags and (if available) gene names from the old D39 annotation (prefix: ‘SPD_’) as query. When identical features were present in R6, a similar search was performed with R6 locus tags (prefix ‘spr’) and gene names. Using the resulting literature, the annotation was further refined.

CDS pseudogenes were detected by performing a BLASTX search against the NCBI non-redundant protein database, using the DNA sequence of two neighboring genes and their intergenic region as query. If the full-length protein was found, the two (or more, after another BLASTX iteration) genes were merged into one pseudogene.

Furthermore, sRNAs and RNA switches, transcriptional start sites and terminators, transcription-regulatory sequences and other useful features (all described below) were added to the annotation. Finally, detected transcript borders (TSSs and terminators) were used to refine coordinates of annotated features (e.g. alternative translational initiation sites). Afterwards, the quality of genome-wide translational initiation site (TIS) calls was evaluated using ‘assess_TIS_annotation.py’ (19).

A total of 178 publications (20–197) was used for curation of the genome annotation.

**Classification of encoded proteins**

Protein sequences corresponding to all CDS features in the final annotation were determined from the corresponding coding sequences. Using InterProScan (198) all proteins were classified according to several databases: TIGRFAMs (199), Gene Ontology (GO, (200, 201)), KEGG (202), InterPro (IPR, (203)), and Protein family (Pfam, (204)).

**Mapping of RNA-seq data**

For the mapping of RNA-seq data, the first 1000 bps of the newly assembled reference genome were copied to the end of the genome, to enable mapping of sequencing reads across the boundaries of the sequence file. Subsequently, we used Bowtie 2 (13) to map Illumina sequencing data (described above) to the extended genome in the appropriate mode (single-end for 5’-enriched, paired-end for control data).

**Normalized start and end counts and complete coverage of sequenced fragments**

The start position of sequenced fragments was extracted from the sequence alignment map (SAM) produced by Bowtie 2. In case of paired-end sequencing, end points of sequenced fragments were also extracted. The positions were used to build strand-specific, single-nucleotide resolution frequency tables (start counts, end counts and coverage). For single-end data, coverage was calculated from the regions covered by the single reads. On the other hand, for paired-end data, the entire inferred fragment (i.e. including the region between mapping sites of mate reads) was used to calculate coverage. Counts of the last 1000 bps of the artificially extended genome were added to those of the first 1000 bps. To obtain normalized values, start counts, end counts and coverage were each divided by the summed genome-wide coverage, excluding positions within 30 nts of rRNA genes.

**Identification of putative coverage termination peaks**

The end count frequency tables for each strand were scanned with window sizes of 3-12 nucleotides and the evaluated window was marked as a candidate termination peak if it met a set of criteria. Firstly, the average end count per nucleotide should be more than 10-fold higher inside the window than in the 8 nucleotides upstream and 8 nucleotides downstream of the window. Secondly, the average number of terminated reads per nucleotide should be higher than 5. Thirdly, peaks were trimmed when the most outer nucleotides had an end frequency of either below 3 or below 1% of the maximum frequency within the window. Then, overlapping candidate peaks were merged and the merged peaks were trimmed as before. The final set of peaks was named ‘putative coverage termination peaks’, shown in PneumoBrowse.

**Detection of small RNA features**

Paired-end sequencing was exploited to detect putative small RNA features. To be conservative, only uniquely mapped read pairs were processed, effectively eliminating reads mapping to rRNA regions. This is the only instance where multi-mappers were excluded. First, to determine the distribution of randomly fragmented transcripts, the sizes of sequenced fragments were extracted from the SAM file corresponding to the paired-end-sequenced control sample. Herein, fragments (partially) mapping within 30 nts upstream of or overlapping with annotated features smaller than 500 nts were excluded. This was done to exclude biases towards lower fragment sizes. In addition, the 10 nts downstream of tRNA genes were excluded, to eliminate fragments derived from tRNA processing. From all remaining fragments, the library-wide size distribution was determined.

Subsequently, for each putative coverage termination peak (see above), fragments from the SAM file that ended inside the peak region were extracted. Of those reads, a peak-specific fragment size distribution was built. A putative sRNA was defined by several criteria: (i) the termination efficiency of the coverage termination peak should be above 30% (see below for the definition of termination efficiency), (ii) the relative frequency of the predicted sRNA length should be more than 25-fold higher than the corresponding frequency in the library-wide distribution, (iii) the predicted sRNA should be completely covered at least 15x for high-confidence (HC) terminators and at least 200x for non-HC terminators (see below for the definition of HC terminators).

The entire process was repeated once more, now also excluding all detected putative sRNAs from the library-wide size distribution. This allowed detection of lower-abundance sRNAs that were of the same size as a highly abundant sRNA identified in the first round. For the scope of this paper, only predicted sRNAs that did not significantly overlap already annotated features were considered.

A candidate sRNA was annotated (either as sRNA or RNA switch) when either (i) a matching entry, with a specified function, was found in RFAM (205) and/or BSRD (150) databases (e.g. T-box leaders, PyrR-binding sites); (ii) the sRNA was validated by Northern blotting in previous studies; or (iii) at least two transcription-regulatory elements were detected (i.e. transcriptional start or termination sites, or sigma factor binding sites).

**Identifying high-confidence terminators**

Since *S. pneumoniae* lacks the Rho factor, all terminators are expected to be ‘Rho-independent’ or intrinsic terminators. These intrinsic terminators consist of a palindromic stem, the arms of which are separated by a small loop, followed by a uracil-rich stretch. Using the new genome assembly as input, putative stem loops followed by U-rich sequences were predicted by TransTermHP (3), with liberal settings to reduce false negative rates: a minimum confidence level of 60, a maximum loop length of 15 (with a penalty increasing linearly with length) and no genomic context restrictions. Subsequently, if a previously identified ‘putative coverage termination peak’ fell inside the poly(U)-tract of a predicted stem-loop structure, the combination of both elements was defined to be a HC-terminator and was included in the genome annotation. Terminator efficiency was determined by the total number of fragments ending in a coverage termination peak, as a percentage of all fragments covering the peak (i.e. including non-terminated fragments).

**Transcription start site identification**

Normalized start counts from 5’-enriched and control libraries were compared. Importantly, normalization was performed excluding reads that mapped to rRNA genes, to account for the fact that the control library was and the 5’-enriched library was not rRNA-depleted. Additionally, all locations within 30 bps of rRNA genes were excluded from further analysis. An initial list was built of unclustered TSSs, which have (i) at least 2.5-fold higher normalized start counts in the 5’-enriched library, compared to the control library, and (ii) a minimum normalized start count of 2 (corresponding to 29 reads) in the 5’-enriched library. Subsequently, TSS candidates closer than 10 nucleotides were clustered, conserving the candidate with the highest start count in the 5’-enriched library. Finally, if the 5’-enriched start count of a candidate TSS was exceeded by the value at the nucleotide immediately upstream, the latter was annotated as TSS instead. The remaining, clustered TSSs are referred to as high-confidence (HC) TSSs.

The 5’-end of some transcripts may be rapidly dephosphorylated, precluding their enrichment in the Cappable-seq approach. Therefore, we included a set of 34 lower confidence (LC) TSSs in our annotation, which were not overrepresented in the 5’-enriched library, but that did meet a set of strict criteria: (i) normalized start count in the control library should be above 10 (corresponding to 222 reads), (ii) a TATAAT motif (with a maximum of 1 mismatch) should be present in the 5-15 nucleotides upstream, (iii) the nucleotide should not be immediately downstream of a processed tRNA, and (iv) the nucleotide should be in an intergenic region. If multiple LC-TSSs were predicted in one intergenic region, only the strongest one was annotated. If a HC-TSS was present in the same intergenic region, the LC-TSS was only annotated when its 5’-enriched start count exceeded that of the HC-TSS.

**TSS classification**

After transcript and CDS boundaries were refined, using the detected transcript boundaries (TSSs and terminators), the final set of high- and low-confidence TSSs were classified into five classes: primary TSSs (only or strongest TSS within 300 nt upstream of a feature), secondary TSSs (within 300 nt upstream of a feature, but not the strongest), internal (inside a feature and on the same strand), antisense (inside a feature or untranslated region, on the opposite strand), and orphan (in none of the other four classes). Antisense TSSs were further divided into 3 subclasses: A_5_ (antisense and upstream of feature), A_3_ (antisense and downstream of feature), and A_0_ (antisense and inside feature).

**Prediction of regulatory motifs**

We proceeded with the identification of binding sites of sigma and transcription factors. First we retrieved reported binding sites for RpoD (σ_A_, (192)), ComX (σ_X_, (72)) and ComE (151), along with pneumococcal extended -10 sequences (206). Using the MEME Suite (118), we built consensus binding motifs: the nucleotide distribution of all regions of 500 nucleotides upstream of TSSs was used as background nucleotide distribution. For RpoD, separate motifs were built for -35 and -10 sequences and five composite motifs were then created with a spacer length of 15-19 nucleotides between the -35 and -10 elements.

Subsequently, using FIMO (207), the 100 nucleotides upstream of each TSS were scanned for a match with the consensus ComE-binding motif (p<0.00001). Similarly, the 40 nucleotides upstream were searched for sites recognized by ComX (p<0.00001) or RpoD (p<0.001). Afterwards, predicted RpoD binding sites were discarded if the spacing between the motif and the corresponding TSS was below 3 or above 8. The spacing between a ComX site and a TSS was required to be below 6. Finally, a search for isolated standard or extended -10 sites was limited to 20 nucleotides upstream of TSSs (p<0.001), again afterwards restricted to be 3-8 nucleotides upstream of the corresponding TSS.

Annotated binding sites of other transcription factors, including CodY and CcpA, were directly adopted from RegPrecise (162).

Ribosomal binding sites (RBSs) were identified by scanning the 16 nucleotides upstream of translation initiation sites (TISs) with MEME, identifying an enriched motif (**Figure 7A**, inset), present for 69% of TISs (p<0.01). Spacing between RBSs and corresponding TISs was restricted to 4-9 nucleotides.

The origin of replication was predicted with OriFinder (106). DnaA-boxes were annotated exclusively in the 5 kbp region surrounding the origin of replication. Perfect matches to the consensus sequence TTWTNCACA (33) were supplemented with single-mismatch sequences located within 5 nucleotides of a perfect match. DnaA-trios were annotated based on sequence identity with their *B. subtilis* counterparts (184).

**Operon prediction and regulon assignment**

Defining an operon as a set of genes controlled by a single promoter, putative operons were predicted for each primary TSS, as defined above. Two consecutive features on the same strand were predicted to be in the same operon if (i) their expression across 22 infection-relevant conditions was strongly correlated (correlation value > 0.75, (4)) and (ii) no strong terminator (>80% efficient) was found between the features. The resulting operons (**Supplementary Table S8**) were allowed to overlap, reflecting the complex nature of the transcriptome. Regulons were assigned according to detected transcription factor sites upstream of primary TSSs, combined with predicted operons.

**Identification of leaderless transcripts**

For all 768 operons starting with a protein-encoding feature (CDS) or pseudogene, the TSS position was compared to the start position of the first feature in the operon. Thereby, 5’-leaders were determined. We revealed 80 operons with a leader of 10 nucleotides or less, while 69 of them had no leader at all.

**Creating PneumoBrowse**

PneumoBrowse (<https://veeninglab.com/pneumobrowse>) is based on JBrowse (208), supplemented with plugins *jbrowse-dark-theme* and *SitewideNotices* (<https://github.com/erasche>), and *ScreenShotPlugin*, *HierarchicalCheckboxPlugin* and *StrandedPlotPlugin* (BioRxiv: <https://doi.org/10.1101/212654>). Annotated elements were divided over five annotation tracks: (i) genes (includes pseudogenes, shown in grey), (ii) putative operons, (iii) regulatory features, including TSSs and terminators, (iv) repeats, and (v) other features. Additionally, full coverage tracks are available, along with start and end counts.

1. Lagousi,T., Routsias,J., Piperi,C., Tsakris,A., Chrousos,G., Theodoridou,M. and Spoulou,V. (2015) Discovery of immunodominant B cell epitopes within surface pneumococcal virulence proteins in pediatric patients with invasive pneumococcal disease. *J. Biol. Chem.*, **290**, 27500–27510.

2. Yun,K.W., Lee,H., Choi,E.H. and Lee,H.J. (2015) Diversity of pneumolysin and pneumococcal histidine triad protein D of *Streptococcus pneumoniae* isolated from invasive diseases in Korean children. *PloS One*, **10**, e0134055.

3. Kingsford,C.L., Ayanbule,K. and Salzberg,S.L. (2007) Rapid, accurate, computational discovery of Rho-independent transcription terminators illuminates their relationship to DNA uptake. *Genome Biol.*, **8**, R22.

4. Aprianto,R., Slager,J. and Veening,J.-W. (2018) High-resolution analysis of the pneumococcal transcriptome under a wide range of infection-relevant conditions. *Nucleic Acids Res.*, **xx**, xx-yy.

5. Aprianto,R., Slager,J., Holsappel,S. and Veening,J.-W. (2016) Time-resolved dual RNA-seq reveals extensive rewiring of lung epithelial and pneumococcal transcriptomes during early infection. *Genome Biol.*, **17**, 198.

6. Gibson,D.G. (2009) Synthesis of DNA fragments in yeast by one-step assembly of overlapping oligonucleotides. *Nucleic Acids Res.*, **37**, 6984–6990.

7. Slager,J., Kjos,M., Attaiech,L. and Veening,J.-W. (2014) Antibiotic-induced replication stress triggers bacterial competence by increasing gene dosage near the origin. *Cell*, **157**, 395–406.

8. Kjos,M., Miller,E., Slager,J., Lake,F.B., Gericke,O., Roberts,I.S., Rozen,D.E. and Veening,J.-W. (2016) Expression of *Streptococcus pneumoniae* bacteriocins is induced by antibiotics via regulatory interplay with the competence system. *PLoS Pathog.*, **12**, e1005422.

9. Ettwiller,L., Buswell,J., Yigit,E. and Schildkraut,I. (2016) A novel enrichment strategy reveals unprecedented number of novel transcription start sites at single base resolution in a model prokaryote and the gut microbiome. *BMC Genomics*, **17**, 199.

10. Barrick,J.E., Colburn,G., Deatherage,D.E., Traverse,C.C., Strand,M.D., Borges,J.J., Knoester,D.B., Reba,A. and Meyer,A.G. (2014) Identifying structural variation in haploid microbial genomes from short-read resequencing data using breseq. *BMC Genomics*, **15**, 1039.

11. Flusberg,B.A., Webster,D.R., Lee,J.H., Travers,K.J., Olivares,E.C., Clark,T.A., Korlach,J. and Turner,S.W. (2010) Direct detection of DNA methylation during single-molecule, real-time sequencing. *Nat. Methods*, **7**, 461–465.

12. Clark,T.A., Murray,I.A., Morgan,R.D., Kislyuk,A.O., Spittle,K.E., Boitano,M., Fomenkov,A., Roberts,R.J. and Korlach,J. (2012) Characterization of DNA methyltransferase specificities using single-molecule, real-time DNA sequencing. *Nucleic Acids Res.*, **40**, e29.

13. Langmead,B. and Salzberg,S.L. (2012) Fast gapped-read alignment with Bowtie 2. *Nat. Methods*, **9**, 357–359.

14. Tatusova,T., DiCuccio,M., Badretdin,A., Chetvernin,V., Ciufo,S. and Li,W. (2013) Prokaryotic Genome Annotation Pipeline. In *The NCBI Handbook*. National Center for Biotechnology Information (US).

15. Overbeek,R., Olson,R., Pusch,G.D., Olsen,G.J., Davis,J.J., Disz,T., Edwards,R.A., Gerdes,S., Parrello,B., Shukla,M., *et al.* (2014) The SEED and the Rapid Annotation of microbial genomes using Subsystems Technology (RAST). *Nucleic Acids Res.*, **42**, D206–D214.

16. Boratyn,G.M., Camacho,C., Cooper,P.S., Coulouris,G., Fong,A., Ma,N., Madden,T.L., Matten,W.T., McGinnis,S.D., Merezhuk,Y., *et al.* (2013) BLAST: a more efficient report with usability improvements. *Nucleic Acids Res.*, **41**, W29–W33.

17. The UniProt Consortium (2017) UniProt: the universal protein knowledgebase. *Nucleic Acids Res.*, **45**, D158–D169.

18. Marchler-Bauer,A. and Bryant,S.H. (2004) CD-Search: protein domain annotations on the fly. *Nucleic Acids Res.*, **32**, W327-331.

19. Overmars,L., Siezen,R.J. and Francke,C. (2015) A novel quality measure and correction procedure for the annotation of microbial translation initiation sites. *PloS One*, **10**, e0133691.

20. Martin,B., Ruellan,J.M., Angulo,J.F., Devoret,R. and Claverys,J.P. (1992) Identification of the *recA* gene of *Streptococcus pneumoniae*. *Nucleic Acids Res.*, **20**, 6412.

21. Martin,C., Briese,T. and Hakenbeck,R. (1992) Nucleotide sequences of genes encoding penicillin-binding proteins from *Streptococcus pneumoniae* and *Streptococcus oralis* with high homology to *Escherichia coli* penicillin-binding proteins 1a and 1b. *J. Bacteriol.*, **174**, 4517–4523.

22. Martin,B., Humbert,O., Camara,M., Guenzi,E., Walker,J., Mitchell,T., Andrew,P., Prudhomme,M., Alloing,G. and Hakenbeck,R. (1992) A highly conserved repeated DNA element located in the chromosome of *Streptococcus pneumoniae*. *Nucleic Acids Res.*, **20**, 3479–3483.

23. Hui,F.M. and Morrison,D.A. (1991) Genetic transformation in *Streptococcus pneumoniae*: nucleotide sequence analysis shows *comA*, a gene required for competence induction, to be a member of the bacterial ATP-dependent transport protein family. *J. Bacteriol.*, **173**, 372–381.

24. Puyet,A., Greenberg,B. and Lacks,S.A. (1990) Genetic and structural characterization of *endA*. *J. Mol. Biol.*, **213**, 727–738.

25. Campa,A.G. de la, Springhorn,S.S., Kale,P. and Lacks,S.A. (1988) Proteins encoded by the DpnI restriction gene cassette. Hyperproduction and characterization of the DpnI endonuclease. *J. Biol. Chem.*, **263**, 14696–14702.

26. Hakenbeck,R., Briese,T. and Ellerbrok,H. (1986) Antibodies against the benzylpenicilloyl moiety as a probe for penicillin-binding proteins. *Eur. J. Biochem. FEBS*, **157**, 101–106.

27. Priebe,S.D., Hadi,S.M., Greenberg,B. and Lacks,S.A. (1988) Nucleotide sequence of the *hexA* gene for DNA mismatch repair in *Streptococcus pneumoniae* and homology of *hexA* to *mutS* of *Escherichia coli* and *Salmonella typhimurium*. *J. Bacteriol.*, **170**, 190–196.

28. Gilbert,J.V., Plaut,A.G., Fishman,Y. and Wright,A. (1988) Cloning of the gene encoding streptococcal immunoglobulin A protease and its expression in *Escherichia coli*. *Infect. Immun.*, **56**, 1961–1966.

29. Walker,J.A., Allen,R.L., Falmagne,P., Johnson,M.K. and Boulnois,G.J. (1987) Molecular cloning, characterization, and complete nucleotide sequence of the gene for pneumolysin, the sulfhydryl-activated toxin of *Streptococcus pneumoniae*. *Infect. Immun.*, **55**, 1184–1189.

30. Larson,T.J., Ludtke,D.N. and Bell,R.M. (1984) sn-Glycerol-3-phosphate auxotrophy of *plsB* strains of *Escherichia coli*: evidence that a second mutation, *plsX*, is required. *J. Bacteriol.*, **160**, 711–717.

31. Sampson,J.S., O’Connor,S.P., Stinson,A.R., Tharpe,J.A. and Russell,H. (1994) Cloning and nucleotide sequence analysis of *psaA*, the *Streptococcus pneumoniae* gene encoding a 37-kilodalton protein homologous to previously reported *Streptococcus* sp. adhesins. *Infect. Immun.*, **62**, 319–324.

32. Martin,B., García,P., Castanié,M.P. and Claverys,J.P. (1995) The *recA* gene of *Streptococcus pneumoniae* is part of a competence-induced operon and controls lysogenic induction. *Mol. Microbiol.*, **15**, 367–379.

33. Schaper,S. and Messer,W. (1995) Interaction of the initiator protein DnaA of *Escherichia coli* with its DNA target. *J. Biol. Chem.*, **270**, 17622–17626.

34. Guenzi,E., Gasc,A.M., Sicard,M.A. and Hakenbeck,R. (1994) A two-component signal-transducing system is involved in competence and penicillin susceptibility in laboratory mutants of *Streptococcus pneumoniae*. *Mol. Microbiol.*, **12**, 505–515.

35. Méjean,V., Salles,C., Bullions,L.C., Bessman,M.J. and Claverys,J.P. (1994) Characterization of the *mutX* gene of *Streptococcus pneumoniae* as a homologue of *Escherichia coli mutT*, and tentative definition of a catalytic domain of the dGTP pyrophosphohydrolases. *Mol. Microbiol.*, **11**, 323–330.

36. Puyet,A. and Espinosa,M. (1993) Structure of the maltodextrin-uptake locus of *Streptococcus pneumoniae*. Correlation to the *Escherichia coli* maltose regulon. *J. Mol. Biol.*, **230**, 800–811.

37. Pestova,E.V., Håvarstein,L.S. and Morrison,D.A. (1996) Regulation of competence for genetic transformation in *Streptococcus pneumoniae* by an auto-induced peptide pheromone and a two-component regulatory system. *Mol. Microbiol.*, **21**, 853–862.

38. Wani,J.H., Gilbert,J.V., Plaut,A.G. and Weiser,J.N. (1996) Identification, cloning, and sequencing of the immunoglobulin A1 protease gene of *Streptococcus pneumoniae*. *Infect. Immun.*, **64**, 3967–3974.

39. Srinivas,P., Kiliç,A.O. and Vijayakumar,M.N. (1997) Site-specific nicking in vitro at *oriT* by the DNA relaxase of Tn5252. *Plasmid*, **37**, 42–50.

40. Grebe,T., Paik,J. and Hakenbeck,R. (1997) A novel resistance mechanism against beta-lactams in *Streptococcus pneumoniae* involves CpoA, a putative glycosyltransferase. *J. Bacteriol.*, **179**, 3342–3349.

41. Stinson,M.W., McLaughlin,R., Choi,S.H., Juarez,Z.E. and Barnard,J. (1998) Streptococcal histone-like protein: primary structure of *hlpA* and protein binding to lipoteichoic acid and epithelial cells. *Infect. Immun.*, **66**, 259–265.

42. Campbell,E.A., Choi,S.Y. and Masure,H.R. (1998) A competence regulon in *Streptococcus pneumoniae* revealed by genomic analysis. *Mol. Microbiol.*, **27**, 929–939.

43. Novak,R., Cauwels,A., Charpentier,E. and Tuomanen,E. (1999) Identification of a *Streptococcus pneumoniae* gene locus encoding proteins of an ABC phosphate transporter and a two-component regulatory system. *J. Bacteriol.*, **181**, 1126–1133.

44. Derré,I., Rapoport,G., Devine,K., Rose,M. and Msadek,T. (1999) ClpE, a novel type of HSP100 ATPase, is part of the CtsR heat shock regulon of *Bacillus subtilis*. *Mol. Microbiol.*, **32**, 581–593.

45. Novak,R., Henriques,B., Charpentier,E., Normark,S. and Tuomanen,E. (1999) Emergence of vancomycin tolerance in *Streptococcus pneumoniae*. *Nature*, **399**, 590–593.

46. Chavagnat,F., Casey,M.G. and Meyer,J. (1999) Purification, characterization, gene cloning, sequencing, and overexpression of aminopeptidase N from *Streptococcus thermophilus* A. *Appl. Environ. Microbiol.*, **65**, 3001–3007.

47. Fernandez-Espla,M.D. and Rul,F. (1999) PepS from *Streptococcus thermophilus*. A new member of the aminopeptidase T family of thermophilic bacteria. *Eur. J. Biochem. FEBS*, **263**, 502–510.

48. García,P., González,M.P., García,E., García,J.L. and López,R. (1999) The molecular characterization of the first autolytic lysozyme of *Streptococcus pneumoniae* reveals evolutionary mobile domains. *Mol. Microbiol.*, **33**, 128–138.

49. Lee,M.S. and Morrison,D.A. (1999) Identification of a new regulator in *Streptococcus pneumoniae* linking quorum sensing to competence for genetic transformation. *J. Bacteriol.*, **181**, 5004–5016.

50. Gibello,A., Collins,M.D., Domínguez,L., Fernández-Garayzábal,J.F. and Richardson,P.T. (1999) Cloning and analysis of the L-lactate utilization genes from *Streptococcus iniae*. *Appl. Environ. Microbiol.*, **65**, 4346–4350.

51. Lange,R., Wagner,C., de Saizieu,A., Flint,N., Molnos,J., Stieger,M., Caspers,P., Kamber,M., Keck,W. and Amrein,K.E. (1999) Domain organization and molecular characterization of 13 two-component systems identified by genome sequencing of *Streptococcus pneumoniae*. *Gene*, **237**, 223–234.

52. Oggioni,M.R. and Claverys,J.P. (1999) Repeated extragenic sequences in prokaryotic genomes: a proposal for the origin and dynamics of the RUP element in *Streptococcus pneumoniae*. *Microbiol. Read. Engl.*, **145 (Pt 10)**, 2647–2653.

53. Palmen,R., Ogunniyi,A.D., Berroy,P., Larpin,S., Paton,J.C. and Trombe,M.C. (1999) Insertional mutation of *orfD* of the DCW cluster of *Streptococcus pneumoniae* attenuates virulence. *Microb. Pathog.*, **27**, 337–348.

54. Rosenow,C., Maniar,M. and Trias,J. (1999) Regulation of the α-galactosidase activity in *Streptococcus pneumoniae*: characterization of the raffinose utilization system. *Genome Res.*, **9**, 1189–1197.

55. Gerdes,K. (2000) Toxin-antitoxin modules may regulate synthesis of macromolecules during nutritional stress. *J. Bacteriol.*, **182**, 561–572.

56. Novak,R., Charpentier,E., Braun,J.S., Park,E., Murti,S., Tuomanen,E. and Masure,R. (2000) Extracellular targeting of choline-binding proteins in *Streptococcus pneumoniae* by a zinc metalloprotease. *Mol. Microbiol.*, **36**, 366–376.

57. Filipe,S.R., Pinho,M.G. and Tomasz,A. (2000) Characterization of the *murMN* operon involved in the synthesis of branched peptidoglycan peptides in *Streptococcus pneumoniae*. *J. Biol. Chem.*, **275**, 27768–27774.

58. Saizieu,A. de, Gardès,C., Flint,N., Wagner,C., Kamber,M., Mitchell,T.J., Keck,W., Amrein,K.E. and Lange,R. (2000) Microarray-based identification of a novel *Streptococcus pneumoniae* regulon controlled by an autoinduced peptide. *J. Bacteriol.*, **182**, 4696–4703.

59. García,E., Llull,D., Muñoz,R., Mollerach,M. and López,R. (2000) Current trends in capsular polysaccharide biosynthesis of *Streptococcus pneumoniae*. *Res. Microbiol.*, **151**, 429–435.

60. Charpentier,E., Novak,R. and Tuomanen,E. (2000) Regulation of growth inhibition at high temperature, autolysis, transformation and adherence in *Streptococcus pneumoniae* by *clpC*. *Mol. Microbiol.*, **37**, 717–726.

61. Adamou,J.E., Heinrichs,J.H., Erwin,A.L., Walsh,W., Gayle,T., Dormitzer,M., Dagan,R., Brewah,Y.A., Barren,P., Lathigra,R., *et al.* (2001) Identification and characterization of a novel family of pneumococcal proteins that are protective against sepsis. *Infect. Immun.*, **69**, 949–958.

62. Quiberoni,A., Biswas,I., Karoui,M.E., Rezaı̈ki,L., Tailliez,P. and Gruss,A. (2001) In vivo evidence for two active nuclease motifs in the double-strand break repair enzyme RexAB of *Lactococcus lactis*. *J. Bacteriol.*, **183**, 4071–4078.

63. Brégeon,D., Colot,V., Radman,M. and Taddei,F. (2001) Translational misreading: a tRNA modification counteracts a +2 ribosomal frameshift. *Genes Dev.*, **15**, 2295–2306.

64. Chastanet,A., Prudhomme,M., Claverys,J.P. and Msadek,T. (2001) Regulation of *Streptococcus pneumoniae clp* genes and their role in competence development and stress survival. *J. Bacteriol.*, **183**, 7295–7307.

65. Bethe,G., Nau,R., Wellmer,A., Hakenbeck,R., Reinert,R.R., Heinz,H.P. and Zysk,G. (2001) The cell wall-associated serine protease PrtA: a highly conserved virulence factor of *Streptococcus pneumoniae*. *FEMS Microbiol. Lett.*, **205**, 99–104.

66. Reichmann,P. and Hakenbeck,R. (2002) A XerD recombinase with unusual active site motifs in *Streptococcus pneumoniae*. *J. Mol. Microbiol. Biotechnol.*, **4**, 101–110.

67. Wagner,C., Saizieu,A. de, Schönfeld,H.-J., Kamber,M., Lange,R., Thompson,C.J. and Page,M.G. (2002) Genetic analysis and functional characterization of the *Streptococcus pneumoniae vic* operon. *Infect. Immun.*, **70**, 6121–6128.

68. Edman,M., Berg,S., Storm,P., Wikström,M., Vikström,S., Öhman,A. and Wieslander,Å. (2003) Structural features of glycosyltransferases synthesizing major bilayer and nonbilayer-prone membrane lipids in *Acholeplasma laidlawii* and *Streptococcus pneumoniae*. *J. Biol. Chem.*, **278**, 8420–8428.

69. Pulliainen,A.T., Haataja,S., Kähkönen,S. and Finne,J. (2003) Molecular basis of H_2_O_2_ resistance mediated by Streptococcal Dpr. Demonstration of the functional involvement of the putative ferroxidase center by site-directed mutagenesis in *Streptococcus suis*. *J. Biol. Chem.*, **278**, 7996–8005.

70. Fadda,D., Pischedda,C., Caldara,F., Whalen,M.B., Anderluzzi,D., Domenici,E. and Massidda,O. (2003) Characterization of *divIVA* and other genes located in the chromosomal region downstream of the *dcw* cluster in *Streptococcus pneumoniae*. *J. Bacteriol.*, **185**, 6209–6214.

71. Täpp,J., Thollesson,M. and Herrmann,B. (2003) Phylogenetic relationships and genotyping of the genus Streptococcus by sequence determination of the RNase P RNA gene, *rnpB*. *Int. J. Syst. Evol. Microbiol.*, **53**, 1861–1871.

72. Dagkessamanskaia,A., Moscoso,M., Hénard,V., Guiral,S., Overweg,K., Reuter,M., Martin,B., Wells,J. and Claverys,J.-P. (2004) Interconnection of competence, stress and CiaR regulons in *Streptococcus pneumoniae*: competence triggers stationary phase autolysis of *ciaR* mutant cells. *Mol. Microbiol.*, **51**, 1071–1086.

73. Kerr,A.R., Adrian,P.V., Estevão,S., de Groot,R., Alloing,G., Claverys,J.-P., Mitchell,T.J. and Hermans,P.W.M. (2004) The Ami-AliA/AliB permease of *Streptococcus pneumoniae* is involved in nasopharyngeal colonization but not in invasive disease. *Infect. Immun.*, **72**, 3902–3906.

74. McAllister,L.J., Tseng,H.-J., Ogunniyi,A.D., Jennings,M.P., McEwan,A.G. and Paton,J.C. (2004) Molecular analysis of the *psa* permease complex of *Streptococcus pneumoniae*. *Mol. Microbiol.*, **53**, 889–901.

75. Brückner,R., Nuhn,M., Reichmann,P., Weber,B. and Hakenbeck,R. (2004) Mosaic genes and mosaic chromosomes–genomic variation in *Streptococcus pneumoniae*. *Int. J. Med. Microbiol.*, **294**, 157–168.

76. Ulijasz,A.T., Andes,D.R., Glasner,J.D. and Weisblum,B. (2004) Regulation of iron transport in *Streptococcus pneumoniae* by RitR, an orphan response regulator. *J. Bacteriol.*, **186**, 8123–8136.

77. Grove,D.E., Willcox,S., Griffith,J.D. and Bryant,F.R. (2005) Differential single-stranded DNA binding properties of the paralogous SsbA and SsbB proteins from *Streptococcus pneumoniae*. *J. Biol. Chem.*, **280**, 11067–11073.

78. Noirclerc-Savoye,M., Le Gouëllec,A., Morlot,C., Dideberg,O., Vernet,T. and Zapun,A. (2005) In vitro reconstitution of a trimeric complex of DivIB, DivIC and FtsL, and their transient co-localization at the division site in *Streptococcus pneumoniae*. *Mol. Microbiol.*, **55**, 413–424.

79. Nováková,L., Sasková,L., Pallová,P., Janeček,J., Novotná,J., Ulrych,A., Echenique,J., Trombe,M.-C. and Branny,P. (2005) Characterization of a eukaryotic type serine/threonine protein kinase and protein phosphatase of *Streptococcus pneumoniae* and identification of kinase substrates. *FEBS J.*, **272**, 1243–1254.

80. Ezraty,B., Dahlgren,B. and Deutscher,M.P. (2005) The RNase Z homologue encoded by *Escherichia coli elaC* gene is RNase BN. *J. Biol. Chem.*, **280**, 16542–16545.

81. Even,S., Pellegrini,O., Zig,L., Labas,V., Vinh,J., Bréchemmier-Baey,D. and Putzer,H. (2005) Ribonucleases J1 and J2: two novel endoribonucleases in *B. subtilis* with functional homology to *E. coli* RNase E. *Nucleic Acids Res.*, **33**, 2141–2152.

82. Guiral,S., Mitchell,T.J., Martin,B. and Claverys,J.-P. (2005) Competence-programmed predation of noncompetent cells in the human pathogen *Streptococcus pneumoniae*: Genetic requirements. *Proc. Natl. Acad. Sci. U. S. A.*, **102**, 8710–8715.

83. Hasona,A., Crowley,P.J., Levesque,C.M., Mair,R.W., Cvitkovitch,D.G., Bleiweis,A.S. and Brady,L.J. (2005) Streptococcal viability and diminished stress tolerance in mutants lacking the signal recognition particle pathway or YidC2. *Proc. Natl. Acad. Sci. U. S. A.*, **102**, 17466–17471.

84. Andersen,E.S., Rosenblad,M.A., Larsen,N., Westergaard,J.C., Burks,J., Wower,I.K., Wower,J., Gorodkin,J., Samuelsson,T. and Zwieb,C. (2006) The tmRDB and SRPDB resources. *Nucleic Acids Res.*, **34**, D163-168.

85. Siguier,P., Perochon,J., Lestrade,L., Mahillon,J. and Chandler,M. (2006) ISfinder: the reference centre for bacterial insertion sequences. *Nucleic Acids Res.*, **34**, D32-36.

86. Nieto,C., Pellicer,T., Balsa,D., Christensen,S.K., Gerdes,K. and Espinosa,M. (2006) The chromosomal *relBE2* toxin–antitoxin locus of *Streptococcus pneumoniae*: characterization and use of a bioluminescence resonance energy transfer assay to detect toxin–antitoxin interaction. *Mol. Microbiol.*, **59**, 1280–1296.

87. Marrer,E., Schad,K., Satoh,A.T., Page,M.G.P., Johnson,M.M. and Piddock,L.J.V. (2006) Involvement of the putative ATP-dependent efflux proteins PatA and PatB in fluoroquinolone resistance of a multidrug-resistant mutant of *Streptococcus pneumoniae*. *Antimicrob. Agents Chemother.*, **50**, 685–693.

88. Pramanik,A. and Braun,V. (2006) Albomycin uptake via a ferric hydroxamate transport system of *Streptococcus pneumoniae* R6. *J. Bacteriol.*, **188**, 3878–3886.

89. Livny,J., Brencic,A., Lory,S. and Waldor,M.K. (2006) Identification of 17 *Pseudomonas aeruginosa* sRNAs and prediction of sRNA-encoding genes in 10 diverse pathogens using the bioinformatic tool sRNAPredict2. *Nucleic Acids Res.*, **34**, 3484–3493.

90. Lu,Y.-J., Zhang,Y.-M., Grimes,K.D., Qi,J., Lee,R.E. and Rock,C.O. (2006) Acyl-phosphates initiate membrane phospholipid synthesis in Gram-positive pathogens. *Mol. Cell*, **23**, 765–772.

91. Crisóstomo,M.I., Vollmer,W., Kharat,A.S., Inhülsen,S., Gehre,F., Buckenmaier,S. and Tomasz,A. (2006) Attenuation of penicillin resistance in a peptidoglycan O-acetyl transferase mutant of *Streptococcus pneumoniae*. *Mol. Microbiol.*, **61**, 1497–1509.

92. Silva,F.J., Belda,E. and Talens,S.E. (2006) Differential annotation of tRNA genes with anticodon CAT in bacterial genomes. *Nucleic Acids Res.*, **34**, 6015–6022.

93. Khoo,S.K., Loll,B., Chan,W.T., Shoeman,R.L., Ngoo,L., Yeo,C.C. and Meinhart,A. (2007) Molecular and structural characterization of the PezAT chromosomal toxin-antitoxin system of the human pathogen *Streptococcus pneumoniae*. *J. Biol. Chem.*, **282**, 19606–19618.

94. Xiao,Z. and Xu,P. (2007) Acetoin metabolism in bacteria. *Crit. Rev. Microbiol.*, **33**, 127–140.

95. Forouhar,F., Kuzin,A., Seetharaman,J., Lee,I., Zhou,W., Abashidze,M., Chen,Y., Yong,W., Janjua,H., Fang,Y., *et al.* (2007) Functional insights from structural genomics. *J. Struct. Funct. Genomics*, **8**, 37–44.

96. Le Bourgeois,P., Bugarel,M., Campo,N., Daveran-Mingot,M.-L., Labonté,J., Lanfranchi,D., Lautier,T., Pagès,C. and Ritzenthaler,P. (2007) The unconventional Xer recombination machinery of Streptococci/Lactococci. *PLoS Genet.*, **3**, e117.

97. Kloosterman,T.G., van der Kooi-Pol,M.M., Bijlsma,J.J.E. and Kuipers,O.P. (2007) The novel transcriptional regulator SczA mediates protection against Zn^2+^ stress by activation of the Zn^2+^-resistance gene *czcD* in *Streptococcus pneumoniae*. *Mol. Microbiol.*, **65**, 1049–1063.

98. Damjanovic,M., Kharat,A.S., Eberhardt,A., Tomasz,A. and Vollmer,W. (2007) The essential *tacF* gene is responsible for the choline-dependent growth phenotype of *Streptococcus pneumoniae*. *J. Bacteriol.*, **189**, 7105–7111.

99. Lux,T., Nuhn,M., Hakenbeck,R. and Reichmann,P. (2007) Diversity of bacteriocins and activity spectrum in *Streptococcus pneumoniae*. *J. Bacteriol.*, **189**, 7741–7751.

100. Halfmann,A., Kovács,M., Hakenbeck,R. and Brückner,R. (2007) Identification of the genes directly controlled by the response regulator CiaR in *Streptococcus pneumoniae*: five out of 15 promoters drive expression of small non-coding RNAs. *Mol. Microbiol.*, **66**, 110–126.

101. Aanensen,D.M., Mavroidi,A., Bentley,S.D., Reeves,P.R. and Spratt,B.G. (2007) Predicted functions and linkage specificities of the products of the *Streptococcus pneumoniae* capsular biosynthetic loci. *J. Bacteriol.*, **189**, 7856–7876.

102. Livny,J., Yamaichi,Y. and Waldor,M.K. (2007) Distribution of centromere-like *parS* sites in bacteria: insights from comparative genomics. *J. Bacteriol.*, **189**, 8693–8703.

103. Huot,J.L., Balg,C., Jahn,D., Moser,J., Emond,A., Blais,S.P., Chênevert,R. and Lapointe,J. (2007) Mechanism of a GatCAB amidotransferase: aspartyl-tRNA synthetase increases its affinity for Asp-tRNA(Asn) and novel aminoacyl-tRNA analogues are competitive inhibitors. *Biochemistry (Mosc.)*, **46**, 13190–13198.

104. Ibrahim,M., Nicolas,P., Bessières,P., Bolotin,A., Monnet,V. and Gardan,R. (2007) A genome-wide survey of short coding sequences in streptococci. *Microbiology*, **153**, 3631–3644.

105. Ueta,M., Ohniwa,R.L., Yoshida,H., Maki,Y., Wada,C. and Wada,A. (2008) Role of HPF (hibernation promoting factor) in translational activity in *Escherichia coli*. *J. Biochem. (Tokyo)*, **143**, 425–433.

106. Gao,F. and Zhang,C.-T. (2008) Ori-Finder: A web-based system for finding *oriC* s in unannotated bacterial genomes. *BMC Bioinformatics*, **9**, 79.

107. Rodionov,D.A., Li,X., Rodionova,I.A., Yang,C., Sorci,L., Dervyn,E., Martynowski,D., Zhang,H., Gelfand,M.S. and Osterman,A.L. (2008) Transcriptional regulation of NAD metabolism in bacteria: genomic reconstruction of NiaR (YrxA) regulon. *Nucleic Acids Res.*, **36**, 2032–2046.

108. Gruber,A.R., Lorenz,R., Bernhart,S.H., Neuböck,R. and Hofacker,I.L. (2008) The Vienna RNA websuite. *Nucleic Acids Res.*, **36**, W70–W74.

109. Denham,E.L., Ward,P.N. and Leigh,J.A. (2008) Lipoprotein signal peptides are processed by Lsp and Eep of *Streptococcus uberis*. *J. Bacteriol.*, **190**, 4641–4647.

110. Attaiech,L., Granadel,C., Claverys,J.-P. and Martin,B. (2008) RadC, a misleading name? *J. Bacteriol.*, **190**, 5729–5732.

111. Paterson,G.K., Nieminen,L., Jefferies,J.M.C. and Mitchell,T.J. (2008) PclA, a pneumococcal collagen-like protein with selected strain distribution, contributes to adherence and invasion of host cells. *FEMS Microbiol. Lett.*, **285**, 170–176.

112. Yamaguchi,M., Terao,Y., Mori,Y., Hamada,S. and Kawabata,S. (2008) PfbA, a novel plasmin- and fibronectin-binding protein of *Streptococcus pneumoniae*, contributes to fibronectin-dependent adhesion and antiphagocytosis. *J. Biol. Chem.*, **283**, 36272–36279.

113. Baur,S., Marles-Wright,J., Buckenmaier,S., Lewis,R.J. and Vollmer,W. (2009) Synthesis of CDP-activated ribitol for teichoic acid precursors in *Streptococcus pneumoniae*. *J. Bacteriol.*, **191**, 1200–1210.

114. Hübscher,J., Lüthy,L., Berger-Bächi,B. and Stutzmann Meier,P. (2008) Phylogenetic distribution and membrane topology of the LytR-CpsA-Psr protein family. *BMC Genomics*, **9**, 617.

115. Rosch,J.W., Gao,G., Ridout,G., Wang,Y.-D. and Tuomanen,E.I. (2009) Role of the manganese efflux system *mntE* for signalling and pathogenesis in *Streptococcus pneumoniae*. *Mol. Microbiol.*, **72**, 12–25.

116. Maruyama,Y., Nakamichi,Y., Itoh,T., Mikami,B., Hashimoto,W. and Murata,K. (2009) Substrate specificity of streptococcal unsaturated glucuronyl hydrolases for sulfated glycosaminoglycan. *J. Biol. Chem.*, **284**, 18059–18069.

117. Kazmierczak,K.M., Wayne,K.J., Rechtsteiner,A. and Winkler,M.E. (2009) Roles of *rel_Spn_* in stringent response, global regulation and virulence of serotype 2 *Streptococcus pneumoniae* D39. *Mol. Microbiol.*, **72**, 590–611.

118. Bailey,T.L., Boden,M., Buske,F.A., Frith,M., Grant,C.E., Clementi,L., Ren,J., Li,W.W. and Noble,W.S. (2009) MEME SUITE: tools for motif discovery and searching. *Nucleic Acids Res.*, **37**, W202-208.

119. Basavanna,S., Khandavilli,S., Yuste,J., Cohen,J.M., Hosie,A.H.F., Webb,A.J., Thomas,G.H. and Brown,J.S. (2009) Screening of *Streptococcus pneumoniae* ABC transporter mutants demonstrates that LivJHMGF, a branched-chain amino acid ABC transporter, is necessary for disease pathogenesis. *Infect. Immun.*, **77**, 3412–3423.

120. Johnsborg,O. and Håvarstein,L.S. (2009) Pneumococcal LytR, a protein from the LytR-CpsA-Psr family, is essential for normal septum formation in *Streptococcus pneumoniae*. *J. Bacteriol.*, **191**, 5859–5864.

121. Turlan,C., Prudhomme,M., Fichant,G., Martin,B. and Gutierrez,C. (2009) SpxA1, a novel transcriptional regulator involved in X-state (competence) development in *Streptococcus pneumoniae*. *Mol. Microbiol.*, **73**, 492–506.

122. Webb,A.J., Karatsa-Dodgson,M. and Gründling,A. (2009) Two-enzyme systems for glycolipid and polyglycerolphosphate lipoteichoic acid synthesis in *Listeria monocytogenes*. *Mol. Microbiol.*, **74**, 299–314.

123. Yesilkaya,H., Spissu,F., Carvalho,S.M., Terra,V.S., Homer,K.A., Benisty,R., Porat,N., Neves,A.R. and Andrew,P.W. (2009) Pyruvate formate lyase is required for pneumococcal fermentative metabolism and virulence. *Infect. Immun.*, **77**, 5418–5427.

124. Geissmann,T., Chevalier,C., Cros,M.-J., Boisset,S., Fechter,P., Noirot,C., Schrenzel,J., François,P., Vandenesch,F., Gaspin,C., *et al.* (2009) A search for small noncoding RNAs in *Staphylococcus aureus* reveals a conserved sequence motif for regulation. *Nucleic Acids Res.*, **37**, 7239–7257.

125. Tsui,H.-C.T., Mukherjee,D., Ray,V.A., Sham,L.-T., Feig,A.L. and Winkler,M.E. (2010) Identification and characterization of noncoding small RNAs in *Streptococcus pneumoniae* serotype 2 strain D39. *J. Bacteriol.*, **192**, 264–279.

126. Fozo,E.M., Makarova,K.S., Shabalina,S.A., Yutin,N., Koonin,E.V. and Storz,G. (2010) Abundance of type I toxin–antitoxin systems in bacteria: searches for new candidates and discovery of novel families. *Nucleic Acids Res.*, **38**, 3743–3759.

127. Denapaite,D., Brückner,R., Nuhn,M., Reichmann,P., Henrich,B., Maurer,P., Schähle,Y., Selbmann,P., Zimmermann,W., Wambutt,R., *et al.* (2010) The genome of *Streptococcus mitis* B6 - what is a commensal? *PLoS ONE*, **5**, e9426.

128. Francl,A.L., Thongaram,T. and Miller,M.J. (2010) The PTS transporters of *Lactobacillus gasseri* ATCC 33323. *BMC Microbiol.*, **10**, 77.

129. Weinberg,Z., Wang,J.X., Bogue,J., Yang,J., Corbino,K., Moy,R.H. and Breaker,R.R. (2010) Comparative genomics reveals 104 candidate structured RNAs from bacteria, archaea, and their metagenomes. *Genome Biol.*, **11**, R31.

130. Jensch,I., Gámez,G., Rothe,M., Ebert,S., Fulde,M., Somplatzki,D., Bergmann,S., Petruschka,L., Rohde,M., Nau,R., *et al.* (2010) PavB is a surface-exposed adhesin of *Streptococcus pneumoniae* contributing to nasopharyngeal colonization and airways infections. *Mol. Microbiol.*, **77**, 22–43.

131. Kumar,R., Shah,P., Swiatlo,E., Burgess,S.C., Lawrence,M.L. and Nanduri,B. (2010) Identification of novel non-coding small RNAs from *Streptococcus pneumoniae* TIGR4 using high-resolution genome tiling arrays. *BMC Genomics*, **11**, 350.

132. Frolet,C., Beniazza,M., Roux,L., Gallet,B., Noirclerc-Savoye,M., Vernet,T. and Di Guilmi,A.M. (2010) New adhesin functions of surface-exposed pneumococcal proteins. *BMC Microbiol.*, **10**, 190.

133. Cohen,S.E., Lewis,C.A., Mooney,R.A., Kohanski,M.A., Collins,J.J., Landick,R. and Walker,G.C. (2010) Roles for the transcription elongation factor NusA in both DNA repair and damage tolerance pathways in *Escherichia coli*. *Proc. Natl. Acad. Sci.*, **107**, 15517–15522.

134. Härtel,T., Klein,M., Koedel,U., Rohde,M., Petruschka,L. and Hammerschmidt,S. (2011) Impact of glutamine transporters on pneumococcal fitness under infection-related conditions. *Infect. Immun.*, **79**, 44–58.

135. Marion,C., Burnaugh,A.M., Woodiga,S.A. and King,S.J. (2011) Sialic acid transport contributes to pneumococcal colonization. *Infect. Immun.*, **79**, 1262–1269.

136. Croucher,N.J., Vernikos,G.S., Parkhill,J. and Bentley,S.D. (2011) Identification, variation and transcription of pneumococcal repeat sequences. *BMC Genomics*, **12**, 120.

137. Barendt,S.M., Sham,L.-T. and Winkler,M.E. (2011) Characterization of mutants deficient in the L,D-carboxypeptidase (DacB) and WalRK (VicRK) regulon, involved in peptidoglycan maturation of *Streptococcus pneumoniae* serotype 2 strain D39. *J. Bacteriol.*, **193**, 2290–2300.

138. Mohammadi,T., van Dam,V., Sijbrandi,R., Vernet,T., Zapun,A., Bouhss,A., Diepeveen-de Bruin,M., Nguyen-Distèche,M., de Kruijff,B. and Breukink,E. (2011) Identification of FtsW as a transporter of lipid-linked cell wall precursors across the membrane. *EMBO J.*, **30**, 1425–1432.

139. Minnen,A., Attaiech,L., Thon,M., Gruber,S. and Veening,J.-W. (2011) SMC is recruited to *oriC* by ParB and promotes chromosome segregation in *Streptococcus pneumoniae*. *Mol. Microbiol.*, **81**, 676–688.

140. Shafeeq,S., Yesilkaya,H., Kloosterman,T.G., Narayanan,G., Wandel,M., Andrew,P.W., Kuipers,O.P. and Morrissey,J.A. (2011) The *cop* operon is required for copper homeostasis and contributes to virulence in *Streptococcus pneumoniae*. *Mol. Microbiol.*, **81**, 1255–1270.

141. Yu,Z., Lavèn,M., Klepsch,M., Gier,J.-W. de, Bitter,W., Ulsen,P. van and Luirink,J. (2011) Role for *Escherichia coli* YidD in membrane protein insertion. *J. Bacteriol.*, **193**, 5242–5251.

142. Marion,C., Aten,A.E., Woodiga,S.A. and King,S.J. (2011) Identification of an ATPase, MsmK, which energizes multiple carbohydrate ABC transporters in *Streptococcus pneumoniae*. *Infect. Immun.*, **79**, 4193–4200.

143. Kloosterman,T.G. and Kuipers,O.P. (2011) Regulation of arginine acquisition and virulence gene expression in the human pathogen *Streptococcus pneumoniae* by transcription regulators ArgR1 and AhrC. *J. Biol. Chem.*, **286**, 44594–44605.

144. Leroux,M., Jia,F. and Szatmari,G. (2011) Characterization of the *Streptococcus suis* XerS recombinase and its unconventional cleavage of the *difSL* site. *FEMS Microbiol. Lett.*, **324**, 135–141.

145. Acebo,P., Martin-Galiano,A.J., Navarro,S., Zaballos,Á. and Amblar,M. (2012) Identification of 88 regulatory small RNAs in the TIGR4 strain of the human pathogen *Streptococcus pneumoniae*. *RNA*, **18**, 530–546.

146. Boutry,C., Wahl,A., Delplace,B., Clippe,A., Fontaine,L. and Hols,P. (2012) Adaptor protein MecA is a negative regulator of the expression of late competence genes in *Streptococcus thermophilus*. *J. Bacteriol.*, **194**, 1777–1788.

147. Mellroth,P., Daniels,R., Eberhardt,A., Rönnlund,D., Blom,H., Widengren,J., Normark,S. and Henriques-Normark,B. (2012) LytA, major autolysin of *Streptococcus pneumoniae*, requires access to nascent peptidoglycan. *J. Biol. Chem.*, **287**, 11018–11029.

148. Mann,B., van Opijnen,T., Wang,J., Obert,C., Wang,Y.-D., Carter,R., McGoldrick,D.J., Ridout,G., Camilli,A., Tuomanen,E.I., *et al.* (2012) Control of virulence by small RNAs in *Streptococcus pneumoniae*. *PLoS Pathog.*, **8**, e1002788.

149. Potter,A.J., Trappetti,C. and Paton,J.C. (2012) *Streptococcus pneumoniae* uses glutathione to defend against oxidative stress and metal ion toxicity. *J. Bacteriol.*, **194**, 6248–6254.

150. Li,L., Huang,D., Cheung,M.K., Nong,W., Huang,Q. and Kwan,H.S. (2013) BSRD: a repository for bacterial small regulatory RNA. *Nucleic Acids Res.*, **41**, D233-238.

151. Martin,B., Soulet,A.-L., Mirouze,N., Prudhomme,M., Mortier-Barrière,I., Granadel,C., Noirot-Gros,M.-F., Noirot,P., Polard,P. and Claverys,J.-P. (2013) ComE/ComE~P interplay dictates activation or extinction status of pneumococcal X-state (competence). *Mol. Microbiol.*, **87**, 394–411.

152. Basavanna,S., Chimalapati,S., Maqbool,A., Rubbo,B., Yuste,J., Wilson,R.J., Hosie,A., Ogunniyi,A.D., Paton,J.C., Thomas,G., *et al.* (2013) The effects of methionine acquisition and synthesis on *Streptococcus pneumoniae* growth and virulence. *PLoS ONE*, **8**, e49638.

153. Shafeeq,S., Kuipers,O.P. and Kloosterman,T.G. (2013) Cellobiose-mediated gene expression in *Streptococcus pneumoniae*: a repressor function of the novel GntR-type regulator BguR. *PloS One*, **8**, e57586.

154. Qaidi,S.E., Yang,J., Zhang,J.-R., Metzger,D.W. and Bai,G. (2013) The vitamin B_6_ biosynthesis pathway in *Streptococcus pneumoniae* is controlled by pyridoxal 5′-phosphate and the transcription factor PdxR and has an impact on ear infection. *J. Bacteriol.*, **195**, 2187–2196.

155. Grigg,J.C., Chen,Y., Grundy,F.J., Henkin,T.M., Pollack,L. and Ke,A. (2013) T box RNA decodes both the information content and geometry of tRNA to affect gene expression. *Proc. Natl. Acad. Sci. U. S. A.*, **110**, 7240–7245.

156. Corrigan,R.M. and Gründling,A. (2013) Cyclic di-AMP: another second messenger enters the fray. *Nat. Rev. Microbiol.*, **11**, 513–524.

157. Laurenceau,R., Péhau-Arnaudet,G., Baconnais,S., Gault,J., Malosse,C., Dujeancourt,A., Campo,N., Chamot-Rooke,J., Le Cam,E., Claverys,J.-P., *et al.* (2013) A type IV pilus mediates DNA binding during natural transformation in *Streptococcus pneumoniae*. *PLoS Pathog.*, **9**, e1003473.

158. Massidda,O., Nováková,L. and Vollmer,W. (2013) From models to pathogens: how much have we learned about *Streptococcus pneumoniae* cell division? *Environ. Microbiol.*, **15**, 3133–3157.

159. Bai,Y., Yang,J., Eisele,L.E., Underwood,A.J., Koestler,B.J., Waters,C.M., Metzger,D.W. and Bai,G. (2013) Two DHH subfamily 1 proteins in *Streptococcus pneumoniae* possess cyclic di-AMP phosphodiesterase activity and affect bacterial growth and virulence. *J. Bacteriol.*, **195**, 5123–5132.

160. Tian,X.-L., Dong,G., Liu,T., Gomez,Z.A., Wahl,A., Hols,P. and Li,Y.-H. (2013) MecA protein acts as a negative regulator of genetic competence in *Streptococcus mutans*. *J. Bacteriol.*, **195**, 5196–5206.

161. Zapun,A., Philippe,J., Abrahams,K.A., Signor,L., Roper,D.I., Breukink,E. and Vernet,T. (2013) In vitro reconstitution of peptidoglycan assembly from the Gram-positive pathogen *Streptococcus pneumoniae*. *ACS Chem. Biol.*, **8**, 2688–2696.

162. Novichkov,P.S., Kazakov,A.E., Ravcheev,D.A., Leyn,S.A., Kovaleva,G.Y., Sutormin,R.A., Kazanov,M.D., Riehl,W., Arkin,A.P., Dubchak,I., *et al.* (2013) RegPrecise 3.0--a resource for genome-scale exploration of transcriptional regulation in bacteria. *BMC Genomics*, **14**, 745.

163. Bai,Y., Yang,J., Zarrella,T.M., Zhang,Y., Metzger,D.W. and Bai,G. (2014) Cyclic Di-AMP impairs potassium uptake mediated by a cyclic di-AMP binding protein in *Streptococcus pneumoniae*. *J. Bacteriol.*, **196**, 614–623.

164. Iannelli,F., Santoro,F., Oggioni,M.R. and Pozzi,G. (2014) Nucleotide sequence analysis of integrative conjugative element Tn5253 of *Streptococcus pneumoniae*. *Antimicrob. Agents Chemother.*, **58**, 1235–1239.

165. Wu,K., Huang,J., Zhang,Y., Xu,W., Xu,H., Wang,L., Cao,J., Zhang,X. and Yin,Y. (2014) A novel protein, RafX, is important for common cell wall polysaccharide biosynthesis in *Streptococcus pneumoniae*: implications for bacterial virulence. *J. Bacteriol.*, **196**, 3324–3334.

166. Manso,A.S., Chai,M.H., Atack,J.M., Furi,L., De Ste Croix,M., Haigh,R., Trappetti,C., Ogunniyi,A.D., Shewell,L.K., Boitano,M., *et al.* (2014) A random six-phase switch regulates pneumococcal virulence via global epigenetic changes. *Nat. Commun.*, **5**, 5055.

167. Afzal,M., Shafeeq,S., Henriques-Normark,B. and Kuipers,O.P. (2015) UlaR activates expression of the *ula* operon in *Streptococcus pneumoniae* in the presence of ascorbic acid. *Microbiology*, **161**, 41–49.

168. Croucher,N.J., Coupland,P.G., Stevenson,A.E., Callendrello,A., Bentley,S.D. and Hanage,W.P. (2014) Diversification of bacterial genome content through distinct mechanisms over different timescales. *Nat. Commun.*, **5**, 5471.

169. Fleurie,A., Lesterlin,C., Manuse,S., Zhao,C., Cluzel,C., Lavergne,J.-P., Franz-Wachtel,M., Macek,B., Combet,C., Kuru,E., *et al.* (2014) MapZ marks the division sites and positions FtsZ rings in *Streptococcus pneumoniae*. *Nature*, **516**, 259–262.

170. Jakob,R.P., Koch,J.R., Burmann,B.M., Schmidpeter,P.A.M., Hunkeler,M., Hiller,S., Schmid,F.X. and Maier,T. (2015) Dimeric structure of the bacterial extracellular foldase PrsA. *J. Biol. Chem.*, **290**, 3278–3292.

171. Chan,W.T., Yeo,C.C., Sadowy,E. and Espinosa,M. (2014) Functional validation of putative toxin-antitoxin genes from the Gram-positive pathogen *Streptococcus pneumoniae*: *phd*-*doc* is the fourth bona-fide operon. *Front. Microbiol.*, **5**, 677.

172. Kurata,T., Nakanishi,S., Hashimoto,M., Taoka,M., Yamazaki,Y., Isobe,T. and Kato,J. (2015) Novel essential gene involved in 16S rRNA processing in *Escherichia coli*. *J. Mol. Biol.*, **427**, 955–965.

173. Afzal,M., Shafeeq,S. and Kuipers,O.P. (2015) Ascorbic acid-dependent gene expression in *Streptococcus pneumoniae* and the activator function of the transcriptional regulator UlaR2. *Front. Microbiol.*, **6**, 72.

174. Afzal,M., Shafeeq,S., Ahmed,H. and Kuipers,O.P. (2015) Sialic acid-mediated gene expression in *Streptococcus pneumoniae* and role of NanR as a transcriptional activator of the *nan* gene cluster. *Appl. Environ. Microbiol.*, **81**, 3121–3131.

175. Dambach,M., Sandoval,M., Updegrove,T.B., Anantharaman,V., Aravind,L., Waters,L.S. and Storz,G. (2015) The ubiquitous *yybP*-*ykoY* riboswitch is a manganese-responsive regulatory element. *Mol. Cell*, **57**, 1099–1109.

176. Price,I.R., Gaballa,A., Ding,F., Helmann,J.D. and Ke,A. (2015) Mn^2+^-sensing mechanisms of *yybP*-*ykoY* orphan riboswitches. *Mol. Cell*, **57**, 1110–1123.

177. Manzoor,I., Shafeeq,S., Afzal,M. and Kuipers,O.P. (2015) Fucose-mediated transcriptional activation of the *fcs* operon by FcsR in *Streptococcus pneumoniae*. *J. Mol. Microbiol. Biotechnol.*, **25**, 120–128.

178. Manzoor,I., Shafeeq,S., Kloosterman,T.G. and Kuipers,O.P. (2015) Co^2+^-dependent gene expression in *Streptococcus pneumoniae*: opposite effect of Mn^2+^ and Co^2+^ on the expression of the virulence genes *psaBCA*, *pcpA*, and *prtA*. *Front. Microbiol.*, **6**, 748.

179. Shoji,T., Takaya,A., Sato,Y., Kimura,S., Suzuki,T. and Yamamoto,T. (2015) RlmCD-mediated U747 methylation promotes efficient G748 methylation by methyltransferase RlmAII in 23S rRNA in *Streptococcus pneumoniae*; interplay between two rRNA methylations responsible for telithromycin susceptibility. *Nucleic Acids Res.*, **43**, 8964–8972.

180. Yunck,R., Cho,H. and Bernhardt,T.G. (2016) Identification of MltG as a potential terminase for peptidoglycan polymerization in bacteria. *Mol. Microbiol.*, **99**, 700–718.

181. Khandokar,Y.B., Srivastava,P., Sarker,S., Swarbrick,C.M.D., Aragao,D., Cowieson,N. and Forwood,J.K. (2016) Structural and functional characterization of the PaaI thioesterase from *Streptococcus pneumoniae* reveals a dual specificity for phenylacetyl-CoA and medium-chain fatty acyl-CoAs and a novel CoA-induced fit mechanism. *J. Biol. Chem.*, **291**, 1866–1876.

182. Escolano-Martínez,M.S., Domenech,A., Yuste,J., Cercenado,M.I., Ardanuy,C., Liñares,J., de la Campa,A.G. and Martin-Galiano,A.J. (2016) DiiA is a novel dimorphic cell wall protein of *Streptococcus pneumoniae* involved in invasive disease. *J. Infect.*, **73**, 71–81.

183. Lowe,T.M. and Chan,P.P. (2016) tRNAscan-SE On-line: integrating search and context for analysis of transfer RNA genes. *Nucleic Acids Res.*, **44**, W54-57.

184. Richardson,T.T., Harran,O. and Murray,H. (2016) The bacterial DnaA-trio replication origin element specifies single-stranded DNA initiator binding. *Nature*, **534**, 412–416.

185. Zheng,J.J., Sinha,D., Wayne,K.J. and Winkler,M.E. (2016) Physiological roles of the dual phosphate transporter systems in low and high phosphate conditions and in capsule maintenance of *Streptococcus pneumoniae* D39. *Front. Cell. Infect. Microbiol.*, **6**, 63.

186. Wu,K., Xu,H., Zheng,Y., Wang,L., Zhang,X. and Yin,Y. (2016) CpsR, a GntR family regulator, transcriptionally regulates capsular polysaccharide biosynthesis and governs bacterial virulence in *Streptococcus pneumoniae*. *Sci. Rep.*, **6**, 29255.

187. Afzal,M., Shafeeq,S., Ahmed,H. and Kuipers,O.P. (2016) N-acetylgalatosamine-mediated regulation of the *aga* operon by AgaR in *Streptococcus pneumoniae*. *Front. Cell. Infect. Microbiol.*, **6**, 101.

188. Fenton,A.K., Mortaji,L.E., Lau,D.T.C., Rudner,D.Z. and Bernhardt,T.G. (2016) CozE is a member of the MreCD complex that directs cell elongation in *Streptococcus pneumoniae*. *Nat. Microbiol.*, **2**, 16237.

189. Al-Bayati,F.A.Y., Kahya,H.F.H., Damianou,A., Shafeeq,S., Kuipers,O.P., Andrew,P.W. and Yesilkaya,H. (2017) Pneumococcal galactose catabolism is controlled by multiple regulators acting on pyruvate formate lyase. *Sci. Rep.*, **7**, 43587.

190. Liu,X., Li,J.-W., Feng,Z., Luo,Y., Veening,J.-W. and Zhang,J.-R. (2017) Transcriptional repressor PtvR regulates phenotypic tolerance to vancomycin in *Streptococcus pneumoniae*. *J. Bacteriol.*, **199**, e00054-17.

191. Liu,X., Gallay,C., Kjos,M., Domenech,A., Slager,J., van Kessel,S.P., Knoops,K., Sorg,R.A., Zhang,J.-R. and Veening,J.-W. (2017) High-throughput CRISPRi phenotyping identifies new essential genes in *Streptococcus pneumoniae*. *Mol. Syst. Biol.*, **13**, 931.

192. Shimada,T., Yamazaki,Y., Tanaka,K. and Ishihama,A. (2014) The whole set of constitutive promoters recognized by RNA polymerase RpoD holoenzyme of *Escherichia coli*. *PloS One*, **9**, e90447.

193. Stamsås,G.A., Straume,D., Ruud Winther,A., Kjos,M., Frantzen,C.A. and Håvarstein,L.S. (2017) Identification of EloR (Spr1851) as a regulator of cell elongation in *Streptococcus pneumoniae*. *Mol. Microbiol.*, **105**, 954–967.

194. Junges,R., Salvadori,G., Shekhar,S., Åmdal,H.A., Periselneris,J.N., Chen,T., Brown,J.S. and Petersen,F.C. (2017) A quorum-sensing system that regulates *Streptococcus pneumoniae* biofilm formation and surface polysaccharide production. *mSphere*, **2**, e00324-17.

195. Zheng,J.J., Perez,A.J., Tsui,H.-C.T., Massidda,O. and Winkler,M.E. (2017) Absence of the KhpA and KhpB (JAG/EloR) RNA-binding proteins suppresses the requirement for PBP2b by overproduction of FtsA in *Streptococcus pneumoniae* D39. *Mol. Microbiol.*, **106**, 793–814.

196. Bonnet,J., Durmort,C., Jacq,M., Mortier-Barrière,I., Campo,N., VanNieuwenhze,M.S., Brun,Y.V., Arthaud,C., Gallet,B., Moriscot,C., *et al.* (2017) Peptidoglycan O-acetylation is functionally related to cell wall biosynthesis and cell division in *Streptococcus pneumoniae*. *Mol. Microbiol.*, **106**, 832–846.

197. Heß,N., Waldow,F., Kohler,T.P., Rohde,M., Kreikemeyer,B., Gómez-Mejia,A., Hain,T., Schwudke,D., Vollmer,W., Hammerschmidt,S., *et al.* (2017) Lipoteichoic acid deficiency permits normal growth but impairs virulence of *Streptococcus pneumoniae*. *Nat. Commun.*, **8**, 2093.

198. Jones,P., Binns,D., Chang,H.-Y., Fraser,M., Li,W., McAnulla,C., McWilliam,H., Maslen,J., Mitchell,A., Nuka,G., *et al.* (2014) InterProScan 5: genome-scale protein function classification. *Bioinforma. Oxf. Engl.*, **30**, 1236–1240.

199. Selengut,J.D., Haft,D.H., Davidsen,T., Ganapathy,A., Gwinn-Giglio,M., Nelson,W.C., Richter,A.R. and White,O. (2007) TIGRFAMs and Genome Properties: tools for the assignment of molecular function and biological process in prokaryotic genomes. *Nucleic Acids Res.*, **35**, D260-264.

200. Ashburner,M., Ball,C.A., Blake,J.A., Botstein,D., Butler,H., Cherry,J.M., Davis,A.P., Dolinski,K., Dwight,S.S., Eppig,J.T., *et al.* (2000) Gene ontology: tool for the unification of biology. The Gene Ontology Consortium. *Nat. Genet.*, **25**, 25–29.

201. The Gene Ontology Consortium (2017) Expansion of the Gene Ontology knowledgebase and resources. *Nucleic Acids Res.*, **45**, D331–D338.

202. Kanehisa,M., Sato,Y., Kawashima,M., Furumichi,M. and Tanabe,M. (2016) KEGG as a reference resource for gene and protein annotation. *Nucleic Acids Res.*, **44**, D457-462.

203. Finn,R.D., Attwood,T.K., Babbitt,P.C., Bateman,A., Bork,P., Bridge,A.J., Chang,H.-Y., Dosztányi,Z., El-Gebali,S., Fraser,M., *et al.* (2017) InterPro in 2017-beyond protein family and domain annotations. *Nucleic Acids Res.*, **45**, D190-199.

204. Finn,R.D., Coggill,P., Eberhardt,R.Y., Eddy,S.R., Mistry,J., Mitchell,A.L., Potter,S.C., Punta,M., Qureshi,M., Sangrador-Vegas,A., *et al.* (2016) The Pfam protein families database: towards a more sustainable future. *Nucleic Acids Res.*, **44**, D279-285.

205. Kalvari,I., Argasinska,J., Quinones-Olvera,N., Nawrocki,E.P., Rivas,E., Eddy,S.R., Bateman,A., Finn,R.D. and Petrov,A.I. (2018) Rfam 13.0: shifting to a genome-centric resource for non-coding RNA families. *Nucleic Acids Res.*, **46**, D335-342.

206. Sabelnikov,A.G., Greenberg,B. and Lacks,S.A. (1995) An extended −10 promoter alone directs transcription of the *dpnII* operon of *Streptococcus pneumoniae*. *J. Mol. Biol.*, **250**, 144–155.

207. Grant,C.E., Bailey,T.L. and Noble,W.S. (2011) FIMO: scanning for occurrences of a given motif. *Bioinforma. Oxf. Engl.*, **27**, 1017–1018.

208. Skinner,M.E., Uzilov,A.V., Stein,L.D., Mungall,C.J. and Holmes,I.H. (2009) JBrowse: a next-generation genome browser. *Genome Res.*, **19**, 1630–1638.
